# Neuron-Derived Estrogen—A Key Neuromodulator in Synaptic Function and Memory

**DOI:** 10.3390/ijms222413242

**Published:** 2021-12-08

**Authors:** Darrell W. Brann, Yujiao Lu, Jing Wang, Gangadhara R. Sareddy, Uday P. Pratap, Quanguang Zhang, Rajeshwar R. Tekmal, Ratna K. Vadlamudi

**Affiliations:** 1 Department of Neuroscience and Regenerative Medicine, Medical College of Georgia, Augusta University, Augusta, GA 30912, USA; JINWANG@augusta.edu; 2 Department of Neurosurgery, Medical College of Georgia, Augusta University, Augusta, GA 30912, USA; yulu@augusta.edu; 3 Department of Obstetrics and Gynecology, University of Texas Health, San Antonio, TX 78229, USA; Sareddy@uthscsa.edu (G.R.S.); pratap@uthscsa.edu (U.P.P.); Tekmal@uthscsa.edu (R.R.T.); vadlamudi@uthscsa.edu (R.K.V.); 4 Department of Neurology, Louisiana State University Health, Shreveport, LA 71103, USA; quanguang.zhang@lsuhs.edu; 5 Audie L. Murphy Division, South Texas Veterans Health Care System, San Antonio, TX 78229, USA

**Keywords:** estradiol, neuroestrogen, neurosteroid, aromatase, synapse, cognition, memory, steroid hormone, neuromodulator

## Abstract

In addition to being a steroid hormone, 17β-estradiol (E_2_) is also a neurosteroid produced in neurons in various regions of the brain of many species, including humans. Neuron-derived E_2_ (NDE_2_) is synthesized from androgen precursors via the action of the biosynthetic enzyme aromatase, which is located at synapses and in presynaptic terminals in neurons in both the male and female brain. In this review, we discuss evidence supporting a key role for NDE_2_ as a neuromodulator that regulates synaptic plasticity and memory. Evidence supporting an important neuromodulatory role of NDE_2_ in the brain has come from studies using aromatase inhibitors, aromatase overexpression in neurons, global aromatase knockout mice, and the recent development of conditional forebrain neuron-specific knockout mice. Collectively, these studies demonstrate a key role of NDE_2_ in the regulation of synapse and spine density, efficacy of excitatory synaptic transmission and long-term potentiation, and regulation of hippocampal-dependent recognition memory, spatial reference memory, and contextual fear memory. NDE_2_ is suggested to achieve these effects through estrogen receptor-mediated regulation of rapid kinase signaling and CREB-BDNF signaling pathways, which regulate actin remodeling, as well as transcription, translation, and transport of synaptic proteins critical for synaptic plasticity and function.

## 1. Introduction

**Figure 1:**
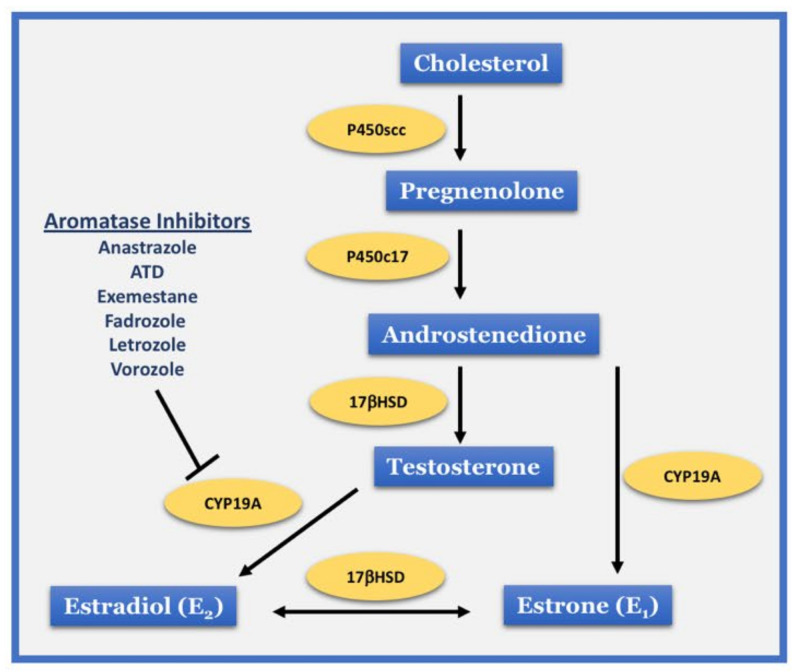
Simplified representation of the pathway for estrogen biosynthesis. Estrogen synthesis begins with conversion of cholesterol to pregnenolone. Through multiple steps, pregnenolone is converted into androstenedione, which is converted into testosterone and estrone (E_1_). Testosterone is then converted into 17β-estradiol (E_2_) through the action of aromatase (CYP19A). Brain aromatase can be inhibited by various aromatase inhibitors.

17β-Estradiol (E_2_) is a steroid hormone synthesized from androgen precursors by the enzyme aromatase (Figure 1), which targets many different organs and tissues in the body, including the central nervous system (CNS) [1]. The neuromodulatory actions of E_2_ in the brain have been widely documented, including regulation of reproduction, sexual behavior, neuroplasticity, and cognition, as well as inflammation and neuroprotection [2,3,4,5,6,7,8,9]. Classically, the female ovary is considered as the main source of circulating E_2_ in the bloodstream. However, there are now abundant studies showing significant expression and activity of aromatase in different brain regions of most species studied to date, including rodents, monkeys, birds, amphibians, and humans [7,10,11,12,13]. Several approaches have been used to study the role of brain-derived estrogen (BDE_2_), including systemic or intracerebral administration of aromatase inhibitors (AI) (Figure 1), intracerebral administration of aromatase antisense oligonucleotides, global aromatase knockout mice, novel forebrain neuron-specific aromatase knockout (FBN-ARO-KO) mice, and astrocyte-specific aromatase knockout (GFAP-ARO-KO) mice. These studies have demonstrated that BDE_2_ regulates many brain functions and processes, including sexual differentiation, reproduction, socio-sexual behavior, synaptic plasticity, cognition, neuroinflammation, and neuroprotection. In this review, we discuss the evidence supporting a role for neuron-derived E_2_ (NDE_2_) in synaptic plasticity and memory.

## 2. Aromatase Localization and Regulation in Neurons

Localization of aromatase in various brain areas has been examined by many groups since the early 1970s through the use of aromatase activity assays, RT-PCR, in situ hybridization, Western blot analysis, immunohistochemistry, and positron emission tomography (PET) imaging. These studies have revealed that, under normal physiological conditions, aromatase is expressed in neurons in many brain regions, including the hippocampus, hypothalamus, amygdala, thalamus, and cortex of both males and females in a variety of species [7,10,11,12,13,14,15,16,17]. In contrast, aromatase is essentially undetectable in astrocytes under basal conditions, but can be induced by cerebral ischemia, trauma, or neuroinflammation [8,18,19,20,21,22]. PET imaging in humans using labeled AI has further confirmed the localization of aromatase in the amygdala, thalamus, preoptic area, hippocampus, cortex, cerebellum, putamen, and white matter of both sexes in humans [23]. Additional work has shown that hippocampal neurons can generate significant quantities of E_2_, as evidenced by concentrations of E_2_ in the rat hippocampus ranging from 1 to 8 nM, which is significantly greater than E_2_ levels in the blood [11,24]. Aromatase has been shown to be localized throughout the neuronal cell body, including in dendrites and axons [15,25], as well as at synapses and presynaptic terminals in many species [11,15,24,26]. The synaptic localization of aromatase has led to the suggestion that NDE_2_ may act as a neurotransmitter/neuromodulator in the brain.

**Figure 2:**
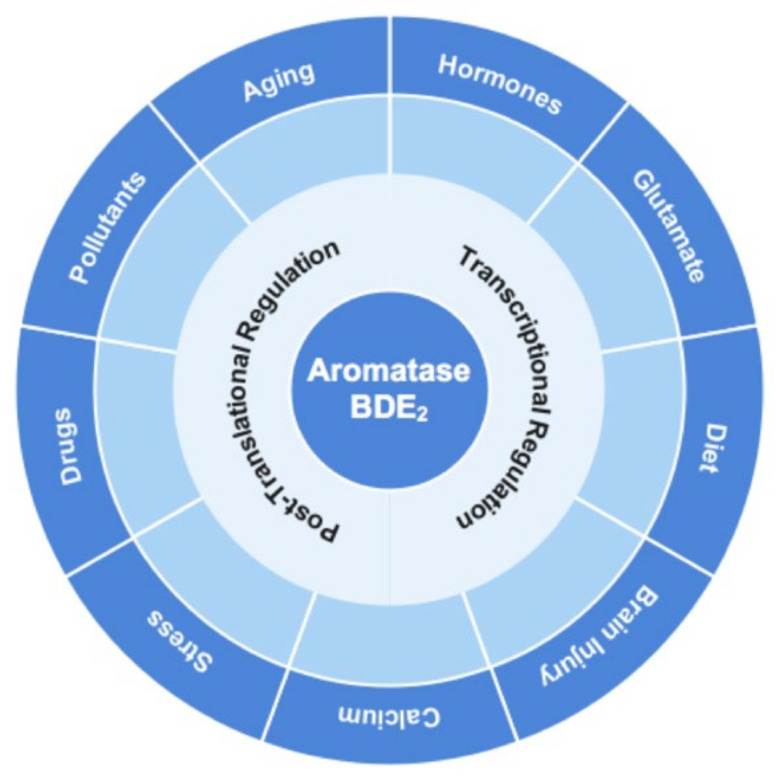
Brain aromatase is regulated by multiple factors. Multiple factors and processes have been implicated to regulate brain aromatase expression and activity. See text for full description and discussion. BDE_2_ = brain-derived 17β-estradiol.

Multiple processes and factors regulate brain aromatase activity and NDE_2_ production (Figure 2).

Phosphorylation of aromatase has been proposed to be a key mechanism for rapid regulation of brain aromatase activity. Human aromatase has 19 putative phosphorylation sites, and aromatase activity in various species can be either increased or decreased rapidly by phosphorylation, with the effect observed depending upon the particular site phosphorylated [27,28,29,30]. Evidence for Ca^2+^-dependent phosphorylation of brain aromatase was first shown in quail and was associated with a rapid decrease of aromatase activity in hypothalamic homogenates and explants [31,32,33]. Furthermore, depletion of Ca^2+^ stores in rat hippocampal neurons and zebra finch forebrain resulted in an increase of E_2_ [34,35]. The neurotransmitter, glutamate has also been shown to rapidly regulate brain aromatase activity and local E_2_ levels, although divergent results have been reported in different species [11,36,37,38]. For instance, administration of glutamate or its agonists rapidly decreased both aromatase activity in quail hypothalamic explants [36], and local E_2_ levels in the caudomedial nidopallium of the zebra finch [37]. In contrast, in vivo administration of the glutamate agonist kainate doubled E_2_ release in the rat hippocampus [38], while the glutamate agonist NMDA strongly increased E_2_ release in rat hippocampal slices in vitro, an effect that was blocked by a NMDA receptor antagonist and dependent upon Ca^2+^ influx [11]. As the major excitatory neurotransmitter in the brain, glutamate regulation of NDE_2_ thus provides a mechanism for rapid changes in NDE_2_ that could serve to facilitate synaptic function and memory, which will be discussed in the next section. Finally, in contrast to glutamate regulation, cholinergic inputs to the hippocampus appear to have no role in the regulation of hippocampal NDE_2_, as cholinergic lesions and administration of cholinesterase inhibitors have no effect upon aromatase expression or activity in the hippocampus [39].

In addition to post-translational regulation, brain aromatase is also regulated at the transcriptional level. Binding sites for many regulatory factors have been identified in the brain aromatase gene, including for transcription factors such as the retinoic acid-related orphan receptor-alpha (RORA) [40], ARP-1 [41], Lhx2 [42], and retinoid X receptor [43]. Knockdown of the ARP-1 or Lhx2 gene in mouse neurons has been shown to significantly decrease aromatase expression [41,42]. Furthermore, overexpression of RORA increased aromatase expression ~10-fold in neuroblastoma cells [40], while treatment with bexarotene, a retinoid X receptor agonist, likewise significantly increased aromatase expression and E_2_ levels in hippocampal slices [43]. Hormones can also regulate aromatase at the transcriptional level. Studies using a mouse hypothalamic neuronal cell line revealed that estrogen receptor α (ERα) interacts with the brain-specific 1.f aromatase promoter, and that E_2_ treatment increases aromatase expression in an ER-dependent manner [44]. Furthermore, testosterone has been shown to increase aromatase mRNA levels and activity in the brain of multiple species [45,46,47,48,49,50]. Interestingly, glucocorticoids have been shown to increase aromatase mRNA and protein levels almost 100-fold in mouse hypothalamic neurons [51]. This may explain why stress, which increases release of the endogenous adrenal glucocorticoid, corticosterone, has likewise been shown to increase aromatase expression and local E_2_ levels in the hypothalamus of female rats [52]. In addition to steroid hormones, both in vitro and in vivo studies have demonstrated that the peptide hormone, the gonadotropin-releasing hormone (GnRH), can also increase E_2_ synthesis in the rat hippocampus [53]. The upregulation of NDE_2_ appears to underlie GnRH effects to enhance synaptic spine density and memory, as administration of letrozole blocks the plasticity and memory-enhancing effects of GnRH [54,55].

There is also evidence that factors such as diet, aging, drugs, and environmental pollutants can regulate brain aromatase. With respect to diet, flavonoids have been suggested to enhance aromatase levels and activity in the brain. Red wine, a well-known flavonoid, increased aromatase expression and activity in the hippocampus following chronic 8-week treatment in rats [56]. On the other hand, phytoestrogens, which are E_2_-like compounds found in plants, were found to have no effect on brain aromatase expression in rats [57,58]. Regarding aging, several studies have found that brain aromatase and E_2_ levels decline with aging. For instance, PET imaging revealed the decreased uptake of the labelled AI, ^11^C-vorozole, in the brains of aged men and women, which suggests that aging decreases aromatase levels in the human brain [59]. Similarly, aged female rats and mice were shown to have a significant reduction in aromatase expression and E_2_ levels in the hippocampus, as compared to young animals [60,61]. With respect to drug regulation of brain aromatase, nicotine has been shown to decrease forebrain aromatase activity in rats [62]. Furthermore, PET imaging using ^11^C-vorozole uptake in the brain suggests that nicotine also decreases aromatase in the non-human primate brain [63,64]. This finding raises the possibility that smoking may decrease brain E_2_ levels, although this remains to be determined. Antipsychotic drugs such as haloperidol and clozapine, but not olanzapine, were also found to decrease aromatase mRNA but not protein levels in the male rat brain after chronic treatment [65]. In contrast, long-term treatment with olanzapine and haloperidol increased aromatase expression in the male rat brainstem [66]. Since antipsychotics can regulate synaptic plasticity [67], it is possible regulation of NDE_2_ could contribute to antipsychotic effects upon synaptic plasticity. However, further studies are needed to measure antipsychotic effects upon local E_2_ levels and examine causality. Finally, a number of studies have found that environmental pollutants such as dioxin, tributyltin, and bisphenol A can increase brain aromatase expression in several species [68,69,70]. The reason for the increase in aromatase expression after environmental pollutant exposure is not clear, but it may be a protective mechanism against neuronal damage by the pollutants, as BDE_2_ has been shown to be neuroprotective against a variety of neural insults [8,20,21,71,72].

## 3. NDE_2_ Is an Important Modulator of Synaptic Plasticity and Cognition

### 3.1. Evidence from Aromatase Inhibitor Studies

Evidence for a role of NDE_2_ in synaptic plasticity was first suggested based on the results of administration of the AI, letrozole, which led to a significant decrease in hippocampal spine synapses and presynaptic boutons in female rat hippocampal slice cultures [73,74]. Additional studies using cultured rat hippocampal neurons confirmed that letrozole decreased spine density in rat hippocampal neurons [73] and in mouse mHippoE-14 hippocampal cells [75]. NDE_2_ may also have a role in preserving dendritic spines and mitochondrial structure in neurodegenerative disorders, as Aβ^1−42^-induced defects in dendritic spines, synaptic proteins, and mitochondrial structure were significantly exacerbated in hippocampal slice cultures when letrozole was combined with Aβ^1−42^ treatment [76]. Interestingly, some in vivo studies found AI effects upon hippocampal spine synapses in females, but not in males [77,78]. For example, systemic treatment with letrozole was reported to decrease hippocampal spine synapses in cycling and ovariectomized female mice and rats, but no significant effect was observed in males [77,78]. However, Zhao et al. [79] reported that letrozole treatment did significantly decrease hippocampal spines, synapses, and synaptic proteins in male mice, and the effect was correlated with a defect in spatial memory. Zhao et. al. [79] used a higher dose of letrozole than the previous studies, which may account for the difference in results.

AI treatment has also been shown to reduce the amplitude of long-term potentiation (LTP) in hippocampal slices from both male and female rats [80,81,82,83], as well as in slices from male rat striatum [84], brainstem [82], and cerebellum [85]. As an electrophysiological parameter of memory function, decreased LTP indicates impaired cognitive function. In support of this, several studies have reported that postmenopausal women with breast cancer who received letrozole treatment displayed a series of memory impairments, including deficits in executive function, processing speed, and verbal and visual learning and memory [86,87]. Furthermore, impairments in hippocampal-dependent memory after AI treatment in women was associated with decreased hippocampal activity during encoding [88]. Similar cognitive defects have also been reported with AI administration in other species. For example, inhibition of hippocampal E_2_ production by central AI administration in adult male zebra finch resulted in worse spatial memory acquisition, along with reduced levels of postsynaptic protein PSD95 [89,90,91]. Likewise, AI treatment in male and female mice and rats was associated with impairment of spatial learning and memory [53,79,92,93]. Additionally, a recent study examining recognition memory in mice found that hippocampal E_2_ levels are elevated within 30 min of novel object training, and letrozole treatment blocked this increase of local E_2_ levels and resulted in impaired hippocampal memory consolidation [94]. Notably, global aromatase knockout mice also display a pronounced cognitive defect, although the actions of ovarian and neuronal-derived estrogen cannot be easily differentiated in this model [95,96].

### 3.2. Evidence from Conditional Knockout Mouse Studies

**Figure 3:**
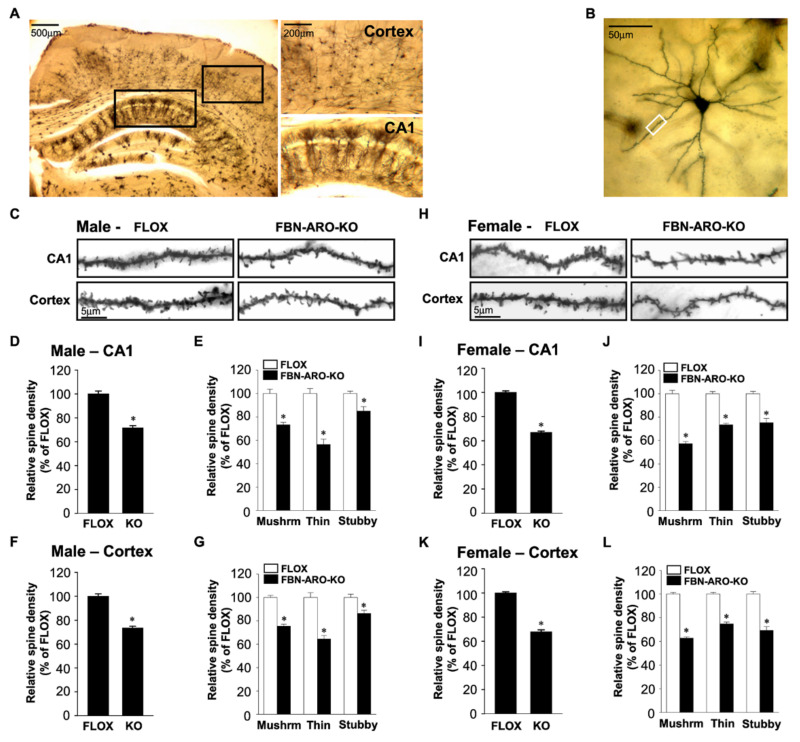
Dendritic spine density was decreased in both male and ovariectomized female FBN-ARO-KO mice. (**A**) Representative images of coronal sections subjected to Golgi staining from the cortex and hippocampal CA1 region. (**B**) Representative single neuron morphology with Golgi staining and the framed apical dendrite section for spine density analysis. (**C**) Representative images of selected dendritic segments from the hippocampal CA1 region and cortex of male FLOX and FBN-ARO-KO mice. (**D**) Quantitative analysis of mean spine density in the hippocampal CA1 region from FLOX and FBN-ARO-KO male mice. (**E**) Quantitative analysis for the changes of different spines, which were classified into mushroom, thin, and stubby based on their morphology. (**F**,**G**) Quantitative analysis of both **(F**) mean spine density and (**G**) different spine morphologies in the cortex of FBN-ARO-KO mice. (**H**) Representative images of dendritic spines from hippocampal CA1 pyramidal and cortical neurons of ovx female FLOX and FBN-ARO-KO mice. (**I**,**J**) Quantitative analysis of (**I**) mean spine density and (**J**) the classified spines in the hippocampal CA1 region from ovx female FLOX and FBN-ARO-KO mice. (**K**,**L**) Group data of (**K**) mean spine density and (**L**) classified spines in cortex of ovx female FLOX and FBN-ARO-KO mice. Values are means ± SEM. *N* = 5. * *p* < 0.05 vs. FLOX group. Mushrm = Mushroom. Adapted from [7].

To extend the previous AI studies and further elucidate the specific roles of NDE_2_ in the forebrain, our group created a forebrain neuron-specific aromatase knockout (FBN-ARO-KO) mouse model using cre-loxP recombination system in technology in which *Cre* is expressed under the control of the CaMKIIα promoter exclusively in forebrain excitatory neurons [7]. The conditional KO mice exhibited a robust 65%–80% decrease of aromatase and E_2_ levels in the hippocampus and cerebral cortex in both sexes, which was specific for the forebrain as there was no change of aromatase expression in the hindbrain or ovaries, and no change in E_2_ serum levels. As shown in Figure 3, detailed examination of FBN-ARO-KO mice revealed a significant decrease in spine density in the hippocampus and cerebral cortex of both intact male and ovariectomized female FBN-ARO-KO mice as compared to FLOX control mice [7].

Interestingly, of the three major spine types (thin, mushroom, and stubby), thin spines showed the highest decrease in the hippocampus and cerebral cortex of male FBN-ARO-KO mice, while mushroom spines were decreased the most in the ovariectomized FBN-ARO-KO female mice. Further work showed that synapse density was also significantly impaired in the hippocampal CA1 region and cerebral cortex of both intact male and ovariectomized female FBN-ARO-KO mice [7].

**Figure 4:**
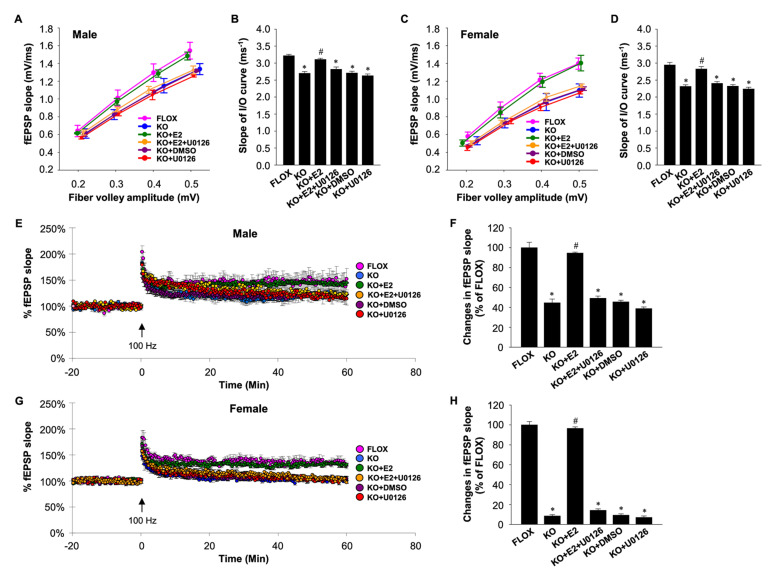
Functional synaptic plasticity is impaired in both male and ovariectomized female FBN-ARO-KO mice and rescued by acute 17β-estradiol (E_2_) treatment. (**A**) Examination of the alteration in excitatory synaptic transmission in male FBN-ARO-KO mice compared with the FLOX control, and the rescue effect by acute E_2_ treatment (1 nM). In another group, U0126 (10 μM), a MEK/ERK inhibitor was co-administered with E_2_ to determine the role of MEK-ERK signaling in the regulation of acute E_2_ benefit on synaptic transmission. (**B**) Quantification of the slopes of I/O curve obtained from linear regression of the I/O curves in A. (**C**,**D**) Analysis of excitatory synaptic transmission in ovariectomized female mice and the rescue effect by acute E_2_ administration (1 nM) on FBN-ARO-KO brain slices. (**C**) In another group, U0126 (10 μM) was co-administered with E_2_ to determine the role of MEK-ERK signaling in E_2_ rescue. (**D**) The corresponding changes in slope of I/O curve were quantified in. (**E**) Long-term potentiation (LTP) recording of Schaffer collateral synapses in above male groups by HFS (100 Hz, 1 s) stimulation, with the fEPSP slope measured for 60 min after LTP induction. (**F**) Quantitative analysis for changes in mean fEPSP slope between 50 and 60 min after LTP induction indicated in E. (**G**) LTP recording in ovariectomized female mice and (**H**) the analysis for fEPSP slope in each group. Values are means ± SEM of determinations from each group. *N* = 6 slices. * *p* < 0.05 vs. FLOX group, *^#^ p* < 0.05 vs. KO group. Adapted from [7].

We further demonstrated that depletion of NDE_2_ in the forebrain of the FBN-ARO-KO male and ovariectomized female mice led to a decrease in efficacy of excitatory synaptic transmission and decreased LTP amplitude in hippocampal slices, which could be rapidly reinstated within minutes by E_2_ replacement [7] (Figure 4). While the decrease in LTP amplitude was highly significant in both sexes, the decrease was greatest in ovariectomized female mice as compared to male mice (e.g., 91% decrease in ovariectomized females versus 57% decrease in males as compared to FLOX controls) [7].

The defect in LTP amplitude in the FBN-ARO-KO mice was correlated with significant defects in hippocampal-dependent spatial memory, recognition memory, and contextual fear memory in intact male, ovariectomized female, and intact female FBN-ARO-KO mice [7]. The cognitive defects appeared to be due to loss of NDE_2_ as they could be rescued by reinstatement of forebrain E_2_ levels in the FBN-ARO-KO mice [7]. In contrast to the role of NDE_2_ in cognitive function, astrocyte-derived E_2_ (ADE_2_) does not appear to have a significant role in learning and memory as we found that male and female astrocyte-specific aromatase knockout (GFAP-ARO-KO) mice had normal hippocampal-dependent spatial and recognition memory and long-term fear memory [20].

## 4. Mechanisms Underlying NDE_2_ Effects on Synaptic Plasticity and Memory

Several mechanisms have been implicated to underlie NDE_2_ regulation of synaptic plasticity and memory, including (1) regulation of actin cytoskeleton polymerization/depolymerization and PSD proteins, (2) enhancement of rapid MAPK/ERK and PI3K-AKT signaling, (3) regulation of CREB-BDNF signaling, and (4) mediation by estrogen receptors and the steroid nuclear receptor co-activator, SRC-1. In support of NDE_2_ regulation of actin polymerization/depolymerization, which is critical for spine formation, several studies found that letrozole treatment in mice resulted in disruption of actin cytoskeleton polymerization dynamics, which was associated with loss of hippocampal spine formation [75,77,97]. Letrozole was also shown to downregulate the actin remodeling proteins profilin-1, phospho-cofilin, and rictor in the mouse hippocampus and in mHippoE-14 hippocampal cells in vitro [75], indicating that NDE_2_ regulates actin polymerization dynamics through regulating expression and/or activity of these key actin remodeling proteins. Furthermore, aromatase overexpression in mHippoE-14 hippocampal cells was shown to induce a significant increase in actin polymerization and the PSD proteins, AMPA GluR1 receptor, and PSD95 [79], further supporting a role of NDE_2_ in regulating actin remodeling and PSD proteins. The aromatase overexpression effects on actin remodeling and PSD proteins were blocked by knockdown of SRC-1, a coactivator of steroid nuclear receptors [75,98], which suggests that the effects of NDE_2_ involve mediation by SRC-1. In addition, the involvement of SRC-1 suggests that steroid nuclear receptors play a role in mediating the plasticity effects of NDE_2_. In support of this suggestion, agonists of estrogen receptor α (ERα) enhanced spine synapse formation in rat hippocampal slices that were treated with letrozole [74]. In contrast, estrogen receptor β (ERβ) agonists reduced spine synapse formation, which suggests a possible antagonistic relationship may exist in the ER subtype control of hippocampal spine synapses [74]. Interestingly, we observed reduced ERα and elevated ERβ levels in the hippocampus and cerebral cortex of FBN-ARO-KO mice, which correlated with reduced spine and synapse density in the hippocampus of these mice [7]. Reinstatement of forebrain E_2_ levels significantly reversed these changes in ERα and ERβ in the hippocampus and cortex of FBN-ARO-KO mice, and rescued defects in synaptic proteins and cognitive function [7]. Since the ER changes in FBN-ARO-KO mice provide only correlational evidence of a role of ERs, more causative-type studies are needed, such as examining rescue effects of ER-selective agonists on synaptic plasticity and memory in FBN-ARO-KO mice.

**Figure 5:**
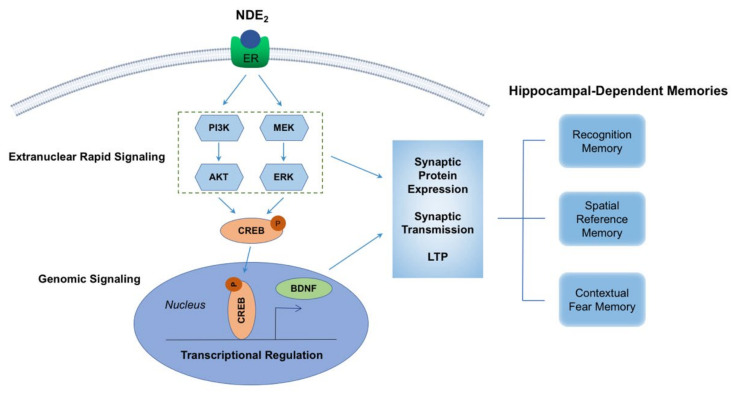
Schematic illustration for the proposed mechanisms underlying NDE_2_ regulation of neuroplasticity and hippocampal-dependent memory. NDE_2_ regulates hippocampal neuroplasticity predominantly through the activation of extranuclear rapid signaling, which includes PI3K-AKT and MEK-ERK pathways. Both PI3K-AKT and MEK/ERK signaling have been implicated in rapidly mediating synaptic plasticity by enhancing synaptic transmission and long-term potentiation (LTP). In addition, PI3K-AKT and MEK/ERK signaling also facilitates the synthesis and transport of synaptic proteins that are necessary for synaptic plasticity. NDE_2_ can also promote CREB activation, which transactivates BDNF transcription via genomic signaling, which can crosstalk with the rapid signaling pathways to mediate synaptic functions and hippocampus-dependent memory, such as recognition memory, spatial reference memory, and contextual fear memory.

Using FBN-ARO-KO mice, we further demonstrated that NDE_2_ is critical for activation of PI3K-AKT and MAPK/ERK rapid kinase signaling in the hippocampus and cerebral cortex of both male and female mice [7]. Furthermore, letrozole treatment has been shown to significantly decrease phospho-AKT levels in the hippocampus of mice, and this effect was reversed by E_2_ replacement [75]. Interestingly, both MAPK/ERK and PI3K-AKT signaling have been implicated to be key mediators of hippocampal neuroplasticity via their roles in the regulation of protein synthesis and transport of synaptic proteins that are necessary for synaptic plasticity [99,100,101,102,103,104]. Thus, NDE_2_ regulation of these rapid kinase signaling pathways is suggested to underlie its plasticity and memory effects. In support of this suggestion, we found that rapid ERK signaling is indeed critical for E_2_ rescue of LTP in FBN-ARO-KO mice, as administration of a MEK-ERK inhibitor blocked the ability of E_2_ to rescue LTP in hippocampal slices from FBN-ARO-KO mice (Figure 4) [7]. In addition, reinstatement of forebrain E_2_ levels rescued defects in phospho-AKT and phospho-ERK levels in the hippocampus and cerebral cortex of FBN-ARO-KO mice, and this effect was correlated with rescue of synaptic density [7]. While NDE_2_ is thought to exert its actions predominantly by extranuclear rapid signaling, findings in FBN-ARO-KO mice demonstrate that NDE_2_ also promotes CREB activation, which transactivates BDNF transcription [7]. As an important neurotrophin, BDNF is critical for synaptic protein expression and regulation of LTP via BDNF-TrkB signaling [105], and knockdown or knockout of BDNF has been shown to impair learning and memory [106,107,108]. While the above implicated signaling pathways could exist in parallel, it is most likely that the pathways crosstalk to mediate NDE_2_ actions and effects in the brain. Indeed, interactions and crosstalk are well known to exist between the pathways. For instance, BDNF is well known to enhance MAPK/ERK and AKT signaling in the brain to help exert its actions, while MAPK/ERK signaling in turn can phosphorylate CREB, which regulates transcription of many genes, including BDNF. A summary diagram of the proposed mechanisms underlying NDE_2_ regulation of synaptic plasticity and memory is provided in Figure 5.

## 5. Conclusions

In conclusion, studies discussed in this paper suggest that NDE_2_ functions as a neuromodulator to regulate neuronal synaptic plasticity and cognitive function. Despite some studies reporting sex differences, increasing evidence supports that NDE_2_ plays similar roles in both the male and female brain. NDE_2_ actions on synaptic plasticity and memory are suggested to be mediated via rapid kinase and CREB-BDNF signaling, which regulates actin remodeling, as well as transcription, translation, and transport of synaptic proteins involved in synaptic plasticity and cognitive function. While much has been learned regarding the roles and actions of NDE_2_ in the brain, much remains to be elucidated. Specifically, it is expected that continued advancement in technology and animal models will allow for a greater understanding of mechanisms underlying regulation of NDE_2_ in the brain as well as how it exerts its key effects on synaptic plasticity and memory. Furthermore, while most studies have focused on excitatory neurons and the hippocampus and cortex, NDE_2_ is also produced in other brain regions including the amygdala, cerebellum, and hypothalamus, as well as in inhibitory neurons, and thus studies to elucidate NDE_2_ roles and actions in these areas and neurons, as well as associated behavioral outcomes, are urgently needed.
